# When the carcinogenic window opens before the screening window: the age-threshold blind spot in early-onset colorectal cancer

**DOI:** 10.3389/fonc.2026.1912787

**Published:** 2026-08-19

**Authors:** Jingtao Zhang, Huanxia Zhou, Guokai Zhang, Hai-mei Wang

**Affiliations:** 1 Linyi Central Hospital, Linyi, Shandong, China; 2 Shandong University of Traditional Chinese Medicine, Jinan, Shandong, China; 3 Binzhou Hospital of Traditional Chinese Medicine, Binzhou, Shandong, China; 4 Department of Oncology, Zibo Central Hospital, Zibo, Shandong, China

**Keywords:** birth cohort effect, cancer screening, carcinogenic window, early-onset colorectal cancer, exposure memory, gut microbiome, risk stratification

## Abstract

Early-onset colorectal cancer (EOCRC) screening is constrained by a structural blind spot concealed by age-threshold–based eligibility. This limitation does not primarily reflect insufficient screening resources, but rather a fundamental temporal mismatch between screening logic and carcinogenic exposure. High-risk states may be established decades earlier through accumulated early-life “exposure memory,” whereas screening triggered by chronological age can intervene only after risk has already been formed. Consequently, individuals younger than 45 years—the population with one of the fastest increases in EOCRC incidence—remain systematically overlooked even after the initiation age for average-risk screening has been lowered to 45 years. This article outlines two parallel pathways: the metabolic–inflammatory axis, including hyperinsulinemia, IGF-1 signaling, and chronic low-grade inflammation; and the gut microbiome axis, including microbial genotoxicity and mucosal immune dysregulation. We clarify how these pathways may encode risk early in life and jointly shift the carcinogenic window forward, making further reductions in age thresholds insufficient for timely identification of truly high-risk young individuals. Accordingly, we argue that EOCRC screening should move from “age-triggered detection” toward “risk-memory recognition.” We propose three strategies stratified by clinical readiness: symptom-triggered colonoscopy triage as diagnostic evaluation rather than screening, earlier assessment of exposure memory, and microbiome-informed risk stratification as a research direction. These approaches may help identify high-risk individuals before the conventional screening age and reduce missed diagnoses and diagnostic delays.

## Introduction

With the widespread use of stool-based testing and colonoscopy, colorectal cancer incidence and mortality among individuals older than 50 years have steadily declined over the past two decades (1). In contrast, among younger adults younger than 50 years, both incidence and risk have shown a stepwise increase across successive birth cohorts. This birth cohort effect cannot be explained solely by calendar-period changes (2) (3). In many countries, the rate and magnitude of increase may already exceed those observed in older populations (4). These patterns suggest that EOCRC may arise from early-life exposures rather than simply representing an earlier onset of tumors traditionally associated with aging (5).

In response, guidelines from the American Cancer Society (ACS) (6), the United States Preventive Services Task Force (USPSTF) (7), and the US Multi-Society Task Force (8) have lowered the recommended starting age for average-risk colorectal cancer screening from 50 to 45 years. This lowering of the screening age represents genuine progress. However, when age is used as the sole trigger for identifying high-risk individuals, chronological age is misread as accumulated “exposure memory.” This conflicts with the definition of exposure memory, namely, a persistent imprint written through epigenetic and immune programming by accumulated metabolic, dietary, and microbial exposures in early life, independent of chronological age itself (3). It therefore creates a fundamental mismatch with the actual temporal distribution of carcinogenic exposure (9, 10), and also conflicts with the established view that risk is shaped by early-life cumulative exposures rather than chronological age per se (11). This mismatch ultimately produces a dual deficit in stratification and screening: individuals at the same chronological age but with distinct exposure histories are classified as having equivalent risk, allowing overscreening in lower-risk individuals and underscreening in higher-risk individuals to coexist (12); meanwhile, individuals younger than 45 years—the group with the fastest increase in incidence—remain outside routine screening eligibility, increasing the risk that symptoms are misclassified as benign because of age anchoring and that diagnosis and treatment are delayed (13).

EOCRC prevention and control involve three distinct clinical contexts: screening in average-risk populations, diagnostic evaluation in symptomatic individuals, and surveillance in individuals with hereditary or otherwise established high-risk conditions. Individuals with hereditary high-risk conditions, such as Lynch syndrome, familial adenomatous polyposis, and inflammatory bowel disease, already follow established surveillance pathways and are therefore not the focus of this article. Instead, we focus on the first two contexts. Within these contexts, young individuals are systematically overlooked in two main ways: first, an omission in screening and risk stratification, whereby asymptomatic individuals with high exposure burden are excluded from average-risk screening because they have not reached the age threshold; and second, a diagnostic omission, whereby symptomatic young individuals are misclassified as having benign conditions because of age anchoring, resulting in delayed diagnostic triage.

**Figure 1:**
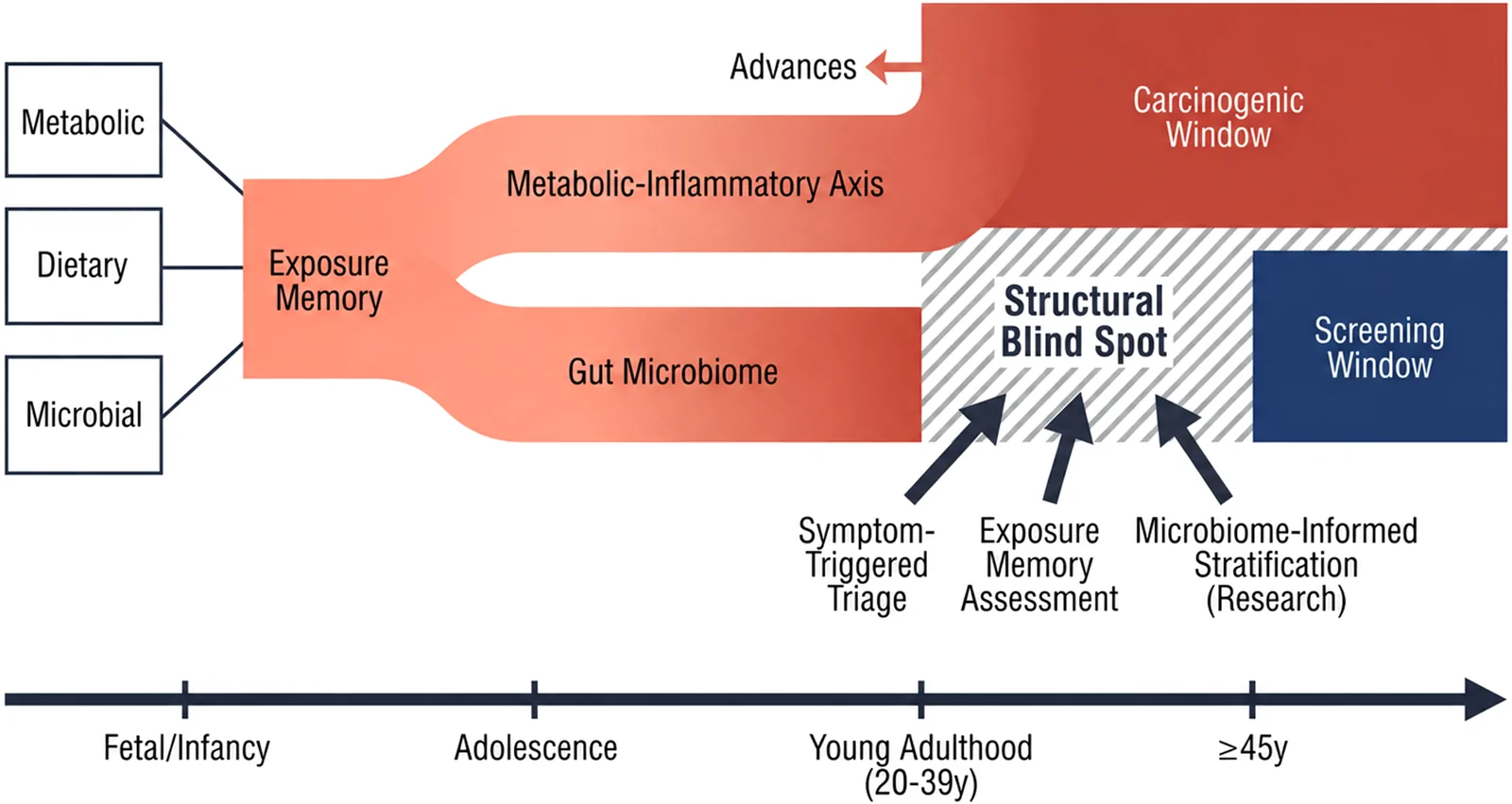
The carcinogenic window advances before the screening window opens: a structural blind spot in age-threshold screening for early-onset colorectal cancer. This image depicts the life-course trajectory of colorectal carcinogenesis from left to right: fetal/infant life, adolescence, young adulthood [20–39 years], and ≥45 years. Early-life metabolic, dietary, and microbial exposures converge through epigenetic and immune programming to establish persistent “exposure memory.” This memory is progressively amplified along two parallel pathways, with the warm color gradient indicating increasing risk over time: the metabolic–inflammatory axis, characterized by hyperinsulinemia, IGF-1 signaling, and chronic low-grade inflammation; and the gut microbial axis, characterized by microbial genotoxicity and mucosal immune dysregulation. Together, these pathways shift the carcinogenic window forward, placing its onset before age 45 and within young adulthood, as indicated by the leftward arrow (“Advances”). In contrast, the current screening window, which is triggered primarily by chronological age, opens only at age 45. The <45-year interval in which carcinogenesis has already begun but screening has not yet been initiated, shown as the hatched region, represents a structural blind spot. This gap reflects a lag in screening logic rather than a mere limitation of resources, resulting in the systematic omission of high-risk individuals under 45 years of age, the group experiencing the fastest increase in early-onset colorectal cancer burden. Accordingly, screening should shift from “age-triggered detection” to “risk-memory recognition,” with earlier recognition and intervention to cover this blind spot through symptom-triggered colonoscopy triage, upstream assessment of exposure memory, and microbiome-informed risk stratification as an emerging research direction, as shown by the three arrows pointing toward the blind spot.

Therefore, we argue that EOCRC screening should shift from “age-triggered detection” to “risk-memory recognition.” Before individuals reach the conventional screening age, high-risk status should be identified according to accumulated early-life exposure trajectories rather than chronological age alone. Corresponding strategies may include symptom-triggered triage, exposure-memory assessment, and microbiome-informed research frameworks, thereby reducing the structural risk of missed diagnoses and diagnostic delays before the routine screening window opens (**Figure 1**).

## Formation of the structural blind spot

The blind spot created by age thresholds is not an incidental omission caused by limited screening resources, but a structural defect that misplaces risk within chronological age. This defect can be analyzed through three interconnected dimensions: the dual omission produced by age-threshold screening, the metabolic–inflammatory axis, and the gut microbiome. Accumulated early-life exposures establish a high-risk state for EOCRC, shift the carcinogenic window forward, and accelerate disease onset, whereas current screening still intervenes only after this window has already opened. As a result, young individuals younger than 45 years are systematically missed.

Although the recommended starting age for average-risk screening has been lowered to 45 years, individuals younger than 45 years remain excluded, and although their absolute incidence remains low (4), EOCRC is not absent in this age group. This gap may continue to widen across successive birth cohorts, and the screening needs of individuals younger than 45 years should therefore not be dismissed. Studies have shown that colorectal cancer incidence is shifting toward younger ages, with steeper increases in younger populations. Incidence among individuals aged 20–29 and 30–39 years has reportedly increased by approximately 7.9% and 4.9% per year, respectively (14).

However, because lowering the screening age to 45 years still fails to cover this population, symptom assessment becomes one of the key tools for early recognition of colorectal cancer. Yet critical early symptoms, including rectal bleeding, abdominal pain, and changes in bowel habits, are often underestimated because of age-related assumptions and limited risk awareness. This constitutes the second mechanism of omission. Young patients may perceive themselves as low risk and delay medical consultation. At the same time, clinicians may anchor on age and misattribute symptoms to benign conditions such as hemorrhoids or irritable bowel syndrome, thereby increasing the risk of misdiagnosis. In addition, the lack of structured triage pathways in routine clinical practice further complicates diagnostic decision-making. Together, these factors may delay the interval from symptom onset to diagnosis by approximately 4–6 months (15), potentially increasing the risk of adverse clinical outcomes.

At the exposure level, cancer risk among individuals younger than 45 years is closely linked to the accumulation of exposure memory and the earlier onset of carcinogenesis. Metabolic and dietary exposures from the fetal period to young adulthood may influence later colorectal cancer risk through epigenetic and immune programming, forming the developmental biological basis of “exposure memory” (3). The rising trajectory of EOCRC overlaps substantially with the global increase in childhood and adolescent obesity and metabolic syndrome (16), suggesting that early-life exposures may exert durable effects on the mucosal microenvironment. Obesity, sugar-sweetened beverage intake (17), and high-fat dietary exposure from early life to young adulthood can induce compensatory hyperinsulinemia and sustained elevation of IGF-1, thereby activating epithelial PI3K/AKT and Wnt/β-catenin proliferative signaling (18, 19). Meanwhile, visceral adipose tissue continuously releases pro-inflammatory mediators such as IL-6 and TNF-α, maintaining the mucosa in a state of oxidative stress and chronic low-grade inflammation. These processes may act synergistically to induce and collectively promote abnormal crypt stem-cell proliferation, inhibit apoptosis, and reshape mucosal immune surveillance from antitumor monitoring toward pro-tumor inflammatory tolerance, and may contribute to an earlier initial age of adenoma formation and increased detection of advanced adenomas among individuals younger than 45 years (20). Studies further indicate that individuals who first become overweight during adolescence or their twenties have a significantly increased risk of early-adult colorectal cancer, and that the association between metabolic abnormalities and EOCRC is stronger in longitudinal assessments than in cross-sectional studies. This supports the view that cancer risk is driven by long-term cumulative exposure rather than by short-term status at a single time point (21, 22). At the molecular level, early-life nutritional exposures may influence later colorectal cancer risk through mitotically transmissible molecular imprints, including DNA methylation and the CpG island methylator phenotype (CIMP) (23, 24). Meanwhile, the gut microbiome may also participate in host epigenetic regulation by inhibiting histone deacetylases (HDACs) through butyrate (25), and by modulating epigenetic enzymes and mucosal immune programming (26).

For EOCRC development, the gut microbiome operates in parallel with the metabolic–inflammatory axis embedded in exposure memory and may contribute to the forward shift of the carcinogenic window. Early-life antibiotic exposure and high consumption of ultra-processed foods can disrupt commensal microbial balance (27, 28), allowing *Escherichia coli* strains carrying the *pks* pathogenicity island and producing colibactin to gain a colonization advantage. Colibactin has been shown in organoid and other models to directly alkylate host epithelial DNA, induce double-strand breaks, and leave highly specific mutational signatures (29). In young epithelial contexts, such damage may facilitate early driver mutations, including *APC* alterations, before damaged clones are effectively repaired or eliminated, thereby shortening the carcinogenic window. An analysis of 981 colorectal cancer genomes from 11 countries showed that the colibactin-associated signatures SBS88 and ID18 were significantly enriched in early-onset colorectal cancer. These signatures were approximately 3.3-fold more frequent in individuals younger than 40 years than in those older than 70 years, and their predominantly early clonal nature suggests that they may be “written” into the genome during early life (30). At the same time, pathogenic microbial communities can remodel mucosal immunity. *Fusobacterium nucleatum* can adhere to and disrupt epithelial tight junctions through Fap2 and FadA, bind inhibitory receptors such as TIGIT on T cells and NK cells, and recruit myeloid-derived suppressor cells, thereby weakening cytotoxic immunity and establishing an immunosuppressive pro-tumor microenvironment (31, 32). Enterotoxigenic *Bacteroides fragilis* can activate STAT3 signaling and IL-17–dependent inflammation through BFT toxin, further amplifying pro-carcinogenic cascades (33). Together, these processes constitute a dual-hit mechanism of “genotoxic mutation plus spatial immune suppression,” promoting the initiation and progression of EOCRC. Because these mechanisms often begin in childhood or adolescence and may persist for years, microbiome-driven immune imbalance should be viewed as a chronic process rather than an acute event. This perspective may partly explain why carcinogenesis begins earlier in young individuals and why colorectal cancer is increasingly observed at younger ages (32).

## Discussion

Although the current age-threshold strategy has lowered the starting age for average-risk colorectal cancer screening to 45 years, three interlinked problems remain unresolved. First, individuals younger than 45 years are still excluded from routine screening, and alarm symptoms in this age group are not consistently managed through structured triage pathways. Second, risk assessment continues to be triggered mainly by chronological age rather than by early-life “exposure memory.” Third, emerging mechanistic evidence, including microbiome-related data, is not yet mature enough to serve as an established stratification dimension. Accordingly, we propose three complementary strategies: symptom-triggered colonoscopy triage, earlier assessment of exposure memory, and microbiome-informed risk stratification. These strategies aim to identify high-risk individuals before the conventional screening age and reduce missed diagnoses and diagnostic delays among people younger than 45 years. This position does not negate the progress achieved by lowering the screening age in current guidelines; rather, it emphasizes that risk identification in younger populations should not be overlooked. The clinical nature, evidence level, clinical readiness, and main limitations of these three recommendations are summarized in **Table 1**. Before these approaches are implemented beyond symptomatic diagnostic triage, their benefit–harm balance should be evaluated against the low absolute risk in individuals younger than 45 years, colonoscopy capacity, false-positive burden, implementation cost, and formal cost-effectiveness.

**Table 1:** Overview of core recommendations.

Recommendation	Clinical nature	Target population	Evidence level	Current status	Main limitations	Ref.
Symptom-triggered colonoscopy triage (± FIT)	Diagnostic evaluation, not screening	Individuals younger than 45 years with alarm symptoms, including rectal bleeding, unexplained iron-deficiency anemia, persistent abdominal pain, or changes in bowel habits	Moderate: evidence supports the diagnostic performance of alarm symptoms and FIT	Implementable at present	Symptom overlap with benign conditions; false positives; colonoscopy capacity; dependence on structured referral pathways	(15, 34)
Exposure-memory risk assessment using standardized questionnaires integrated into electronic health records	Risk stratification to refine screening eligibility	Asymptomatic individuals younger than 45 years with cumulative early-life exposures	Weak to exploratory: no prospectively validated composite score is currently available	Requires prospective validation	Uncertain weighting; recall bias in early-life exposure data; unproven incremental value beyond age; implementation burden	(36, 37)
Microbiome-informed stratification using metagenomics, colibactin-related markers, *Fusobacterium nucleatum* burden, and FIT	Research direction	Asymptomatic young cohorts in research settings	Hypothesis-generating: based mainly on retrospective genomics and preclinical evidence	Not yet clinically deployable	Not prospectively validated in precancerous lesions or asymptomatic cohorts; not incorporated into guidelines; cross-ethnic stability and incremental PPV remain unquantified	(32, 39)

For evidence level, “moderate” indicates relatively consistent observational or translational evidence; “weak” indicates limited or indirect evidence; and “hypothesis-generating” indicates biological plausibility without sufficient validation. The related mechanistic pathways and stratification tools remain at the research stage and require further validation and optimization through prospective cohorts and randomized controlled trials.

Because the birth cohort effect is still largely described at the epidemiological level, clinical tools are needed to capture cohort-specific risk in individuals younger than 45 years and to translate alarm symptoms into actionable stratification variables. We suggest that patients who have not yet reached the routine screening age but present with key early symptoms—such as rectal bleeding, unexplained iron-deficiency anemia, persistent abdominal pain, or changes in bowel habits—should be managed through symptom-triggered colonoscopy triage which constitutes diagnostic evaluation rather than screening and is the most immediately actionable of the three proposed strategies, rather than being presumed to have benign disease. Specifically, alarm symptoms combined with fecal immunochemical testing (FIT) could be used for initial stratification. FIT has shown good performance for identifying colorectal cancer in symptomatic populations (34). Patients with a positive FIT result or multiple alarm symptoms should undergo expedited colonoscopy to minimize diagnostic delay (13, 35). This approach may provide a more practical safeguard for colorectal health in individuals younger than 45 years.

Current CRC risk models rarely incorporate longitudinal exposure trajectories, and “exposure memory” has not yet been translated into a prospectively validated composite score. We therefore recommend developing standardized questionnaires and incorporating key exposure-memory variables into electronic health records, including early-life obesity and weight trajectory (16), antibiotic exposure history (27), high-sugar and high-fat dietary patterns (28), sedentary behavior, prediabetes, and insulin resistance. This would enable earlier risk identification for young-onset CRC and support more timely prevention and intervention. The work by Wehbe et al., which used cumulative risk factors for EOCRC modeling, provides a relevant methodological reference (36). A two-step strategy could be adopted: first, routinely collect readily available indicators such as BMI across life stages, metabolic syndrome components, family history, and smoking status; second, supplement these data with questionnaire-based or interview-based information on early-life exposures and assign weighted scores to generate a composite risk score. This would upgrade the current “age-as-risk” screening logic into a more precise and granular system based on cumulative exposure stratification (37). High-risk young individuals could then be pre-emptively flagged for targeted prevention, shared decision-making, and prospective risk-adapted follow-up in validation studies, rather than immediate routine colonoscopic surveillance.

Microbiome-related evidence remains largely derived from retrospective tissue genomics, *in vitro* studies, and animal experiments. It has not yet been prospectively validated in precancerous lesions or asymptomatic young cohorts, nor has it been incorporated as an independent dimension in authoritative guidelines. Therefore, microbiome-informed risk stratification should currently be positioned as a research pathway rather than an immediately deployable clinical tool. After high-quality evidence from prospective cohorts and randomized studies is established, it may be considered as a complementary component of risk assessment. Drawing on the successful development of stool-based molecular screening (38), microbiome data could be evaluated in prospective cohorts as an incremental supplement to FIT and stool DNA testing, rather than as a direct replacement. In asymptomatic young and middle-aged cohorts, stool metagenomics, colibactin-related markers, *Fusobacterium nucleatum* burden, and systemic inflammatory indicators could be collected in parallel and integrated with quantitative FIT. Machine-learning approaches could then be used to derive composite “inflammation-plus-mutation” markers, thereby generating prospective evidence for future incorporation of microbiome features into risk stratification. During this process, the incremental positive predictive value over existing tools and the cross-ethnic stability of these markers should be quantitatively evaluated to ensure their reliability and generalizability (32, 39).

## Conclusion

Lowering the starting age for colorectal cancer screening from 50 to 45 years represents an important advance. However, the trigger framework underlying current screening logic still has a critical limitation. Age thresholds remain clinically practical as operational starting points, but when they are used as the sole trigger for identifying high-risk individuals, the true risk shaped by early-life cumulative exposures across birth cohorts is incorrectly aligned with chronological age. As a result, individuals younger than 45 years—the group with one of the fastest increases in incidence—remain positioned before the screening window opens. The resulting missed diagnoses and diagnostic delays therefore reflect not only insufficient screening coverage, but also a fundamental mismatch between screening logic and the temporal biology of carcinogenic exposure. The key to addressing the EOCRC screening dilemma is not simply to continue lowering the age threshold, but to supplement age-triggered detection with “risk-memory recognition”: timely diagnostic triage for symptomatic individuals and earlier risk stratification for those with high exposure burden, thereby creating opportunities for preventable early intervention in young populations before the carcinogenic window has already shifted forward.

## Data Availability

The original contributions presented in the study are included in the article/supplementary material. Further inquiries can be directed to the corresponding author.
